# Investigating the relevance of major signaling pathways in cancer survival using a biologically meaningful deep learning model

**DOI:** 10.1186/s12859-020-03850-6

**Published:** 2021-02-05

**Authors:** Jiarui Feng, Heming Zhang, Fuhai Li

**Affiliations:** 1 Institute for Informatics (I2), Washington University School of Medicine, Washington University in St. Louis, St. Louis, MO USA; 2 Data Science, Washington University School of Medicine, Washington University in St. Louis, St. Louis, MO USA; 3 Computer Science, Washington University School of Medicine, Washington University in St. Louis, St. Louis, MO USA; 4 Department of Pediatrics, Washington University School of Medicine, Washington University in St. Louis, St. Louis, MO USA

**Keywords:** Deep learning, Survival analysis, Signaling pathways, TCGA

## Abstract

**Background:**

Survival analysis is an important part of cancer studies. In addition to the existing Cox proportional hazards model, deep learning models have recently been proposed in survival prediction, which directly integrates multi-omics data of a large number of genes using the fully connected dense deep neural network layers, which are hard to interpret. On the other hand, cancer signaling pathways are important and interpretable concepts that define the signaling cascades regulating cancer development and drug resistance. Thus, it is important to investigate potential associations between patient survival and individual signaling pathways, which can help domain experts to understand deep learning models making specific predictions.

**Results:**

In this exploratory study, we proposed to investigate the relevance and influence of a set of core cancer signaling pathways in the survival analysis of cancer patients. Specifically, we built a simplified and partially biologically meaningful deep neural network, DeepSigSurvNet, for survival prediction. In the model, the gene expression and copy number data of 1967 genes from 46 major signaling pathways were integrated in the model. We applied the model to four types of cancer and investigated the influence of the 46 signaling pathways in the cancers. Interestingly, the interpretable analysis identified the distinct patterns of these signaling pathways, which are helpful in understanding the relevance of signaling pathways in terms of their application to the prediction of cancer patients’ survival time. These highly relevant signaling pathways, when combined with other essential signaling pathways inhibitors, can be novel targets for drug and drug combination prediction to improve cancer patients’ survival time.

**Conclusion:**

The proposed DeepSigSurvNet model can facilitate the understanding of the implications of signaling pathways on cancer patients’ survival by integrating multi-omics data and clinical factors.

## Background

Survival analysis based on clinical factors (e.g., age, gender, race, stage) is crucial for cancer prognosis. However, it is just as important to identify and understand essential biomarkers given large-scale genomics data (e.g., gene expression and copy number variation). The cox proportional hazards model (Cox PH) model [1] is the classic model for survival analysis. The Kaplan–Meier estimator curve [2], CoxPH model and logrank test [3] are widely used to display and compare the survival probability over time of patients in different groups or conditions. Recently, deep learning models have been developed for survival analysis. Deep learning models have been used widely in image analysis [4, 5], medical informatics data analysis [6], and natural language process (NLP) [7], and have shown exceptional performance over traditional machine learning models. Thus, deep learning models developed for survival analysis are also promising, e.g., DeepSurv [8], Cox-nnet [9], SCNN [10], and DeepHit [11].

Compared with the Cox PH model, the deep learning models showed improved prediction accuracy by flexibly integrating a large number of genomics features without strong parametric assumptions. For example, the DeepSurv [8] model used the deep neural network to integrate the biomarker genes and personal treatment information to improve the survival time prediction. The DeepHit [11] model also used a deep neural network, and jointly model different events, like different causes of death. In the liver cancer subtyping and survival analysis [12], the auto-encoder model was first employed to reduce the dimensions of the feature space given the large-number of genomics features (e.g., gene expression, miRNA, methylation). The important features (non-linear combinations of raw genomics features) were identified using the Cox PH model [1] for clustering analysis which identified sub-groups with distinct survival outcomes. Then, the analysis of variance (ANOVA) based on the clustering results was applied to the raw genomics features to further identify the important genes. However, the auto-encoder model itself was not used to identify the important raw genomics features in a non-linear perspective. In the Cox-nnet model [9], RNA-seq data from The Cancer Genome Atlas (TCGA) samples was used as the input in a deep neural network to predict the survival time. To identify the potentially associated signaling pathways of hidden nodes, the Pearson’s correlation values between the expression of individual genes and the output of the given hidden nodes were calculated to identify the most linearly correlated genes. Then, gene set enrichment analysis (GSEA) [13] was employed to link the hidden nodes with the enriched signaling pathways. Moreover, the Survival Convolutional Neural Networks (SCNN) [10] was developed to predict survival using histologic images of cancer patients. Finally, heat map visualizations of the regions of interest (image patches) from the SCNN model output were overlaid on the image to indicate the significant regions in the images correlated with survival outcome.

Compared with existing models, we aimed to investigate the relevance or influence of individual cancer signaling pathways (pathway level) to the survival time prediction in cancer patients. In another word, instead of using multi-omics data of a large number of genes, a set of cancer signaling pathways were modeled using a simplified and partially biological meaningful deep neural network architecture, which has not been well investigated. In cancer studies, many dysfunctional signaling pathways that play important roles in tumor development and drug response are identified. For example, the analysis of ten signaling pathways using the TCGA cancer samples indicated that many genetic biomarkers were included in the ten signaling pathways [14]. Such cancer signaling pathways and cancer hallmark networks have been used for prediction of cancer clinical phenotypes and cancer prognosis [15, 16]. In this study, we aimed to investigate the relevance or influence of these signaling pathways within the context of survival outcome prediction using a biologically meaningful and simplified deep learning model, DeepSigSurvNet. Specifically, only signaling pathways (46 pathways) were collected from the KEGG [17] signaling database. The gene expression and copy number data of 1967 genes from the 46 major signaling pathways are from four types of cancer: breast invasive carcinoma (BRCA), lung adenocarcinoma (LUAD), glioblastoma multiforme (GBM), and skin cutaneous melanoma (SKCM). The model was evaluated using the c-index. Moreover, it is critical that domain experts can understand the mechanisms of deep learning models making specific predictions. It is challenging because the complex network architectures. To interpret deep learning models’ prediction, a set of interpretation and explaining approaches have been proposed, e.g., the smmothgrad [18] and Layer-Wise Relevance Propagation (LRP) approach [19], to identify the features that can influence the model prediction results. Interestingly, the interpretable analysis using the smoothgrad approach identified distinct probability density distribution patterns of these signaling pathways, which can be helpful in understanding the relevance of the signaling pathways in terms of their association with cancer patients’ survival. These important signaling pathways can be novel targets for drug and drug combination prediction to improve cancer patients’ survival time. In the following sections, the materials and methods, results and discussions are presented.

## Methods

### RNA-seq and Copy number data of 4 types of cancer

From the UCSC Xena data server, the mean-normalized log2 scaled RSEM [20] values (per gene) across all TCGA cohorts (HiSeqV2_PANCAN dataset) and integer copy number data (per gene) from GISTIC2 analysis were downloaded for four types of cancer: breast invasive carcinoma (BRCA), lung adenocarcinoma (LUAD), glioblastoma multiforme (GBM), and skin cutaneous melanoma (SKCM). The phenotype (clinical) data (survival time, age, gender, stage, etc.) of the cancer samples are also available from the Xena data server. Table 1 shows the number of cancer samples, dataset and URLs to download these datasets. For the purposes of prediction, cancer patients with survival times greater than 3000 days are not included.

**Table 1:** Number of samples, dataset_id and URLs to download the gene expression and copy number data from UCSC Xena data server

Cancer type	DataSet	URLs
BRCA (n = 1057)	HiSeqV2_PANCAN	https://xenabrowser.net/datapages/?cohort=TCGA%20Breast%20Cancer%20(BRCA)&removeHub=https%3A%2F%2Fxena.treehouse.gi.ucsc.edu%3A443
	Gistic2_CopyNumber_Gistic2_all_thresholded.by_genes	
LUAD (n = 500)	HiSeqV2_PANCAN	https://xenabrowser.net/datapages/?cohort=TCGA%20Lung%20Adenocarcinoma%20(LUAD)&removeHub=https%3A%2F%2Fxena.treehouse.gi.ucsc.edu%3A443
	Gistic2_CopyNumber_Gistic2_all_thresholded.by_genes	
GBM (n = 484)	HiSeqV2_PANCAN	https://xenabrowser.net/datapages/?cohort=TCGA%20Glioblastoma%20(GBM)&removeHub=https%3A%2F%2Fxena.treehouse.gi.ucsc.edu%3A443
	Gistic2_CopyNumber_Gistic2_all_thresholded.by_genes	
SKCM (n = 358)	HiSeqV2_PANCAN	https://xenabrowser.net/datapages/?cohort=TCGA%20Melanoma%20(SKCM)&removeHub=https%3A%2F%2Fxena.treehouse.gi.ucsc.edu%3A443
	Gistic2_CopyNumber_Gistic2_all_thresholded.by_genes	

### The 46 major signaling pathways

KEGG (Kyoto Encyclopedia of Genes and Genomes) [17] is a database for the systematic understanding of gene functions. The KEGG signaling pathways provide knowledge of signaling transduction and cellular processes. There are 303 pathways in the KEGG database, and 45 of them are annotated as “signaling pathways”. Many of the signaling pathways are important oncogenic signaling pathways [14], e.g., EGFR, WNT, Hippo, Notch, PI3K-Akt, RAS, TGFβ, p53. The ‘cell cycle’ cellular process is also included. For simplicity, the ‘cell cycle’ is also viewed as one ‘signaling’ pathway. In total, 46 signaling pathways (45 signaling pathways + cell cycle) are selected (see Table 2). Among these 46 signaling pathways, there are 1967 genes with both gene expression and copy number variation data. In summary, there are gene expression (TPM) and copy number variation data of 1967 genes in 46 signaling pathways of 45 cancer cell lines, which was used as the input for the deep learning model.

**Table 2:** The 46 signaling pathways used for analysis

MAPK	FoxO	TGF-beta	T cell receptor	Adipocytokine
ErbB	Sphingolipid	VEGF	B cell receptor	Oxytocin
Ras	Phospholipase D	Apelin	Fc epsilon RI	Glucagon
Rap1	p53	Hippo	TNF	Relaxin
Calcium	mTOR	Toll-like receptor	Neurotrophin	AGE-RAGE
cGMP-PKG	PI3K-Akt	NOD-like receptor	Insulin	Cell cycle
cAMP	AMPK	RIG-I-like receptor	GnRH	
Chemokine	Wnt	C-type lectin receptor	Estrogen	
NF-kappa B	Notch	JAK-STAT	Prolactin	
HIF-1	Hedgehog	IL-17	Thyroid hormone	

### Model Architecture of DeepSigSurvNet

**Fig. 1:**
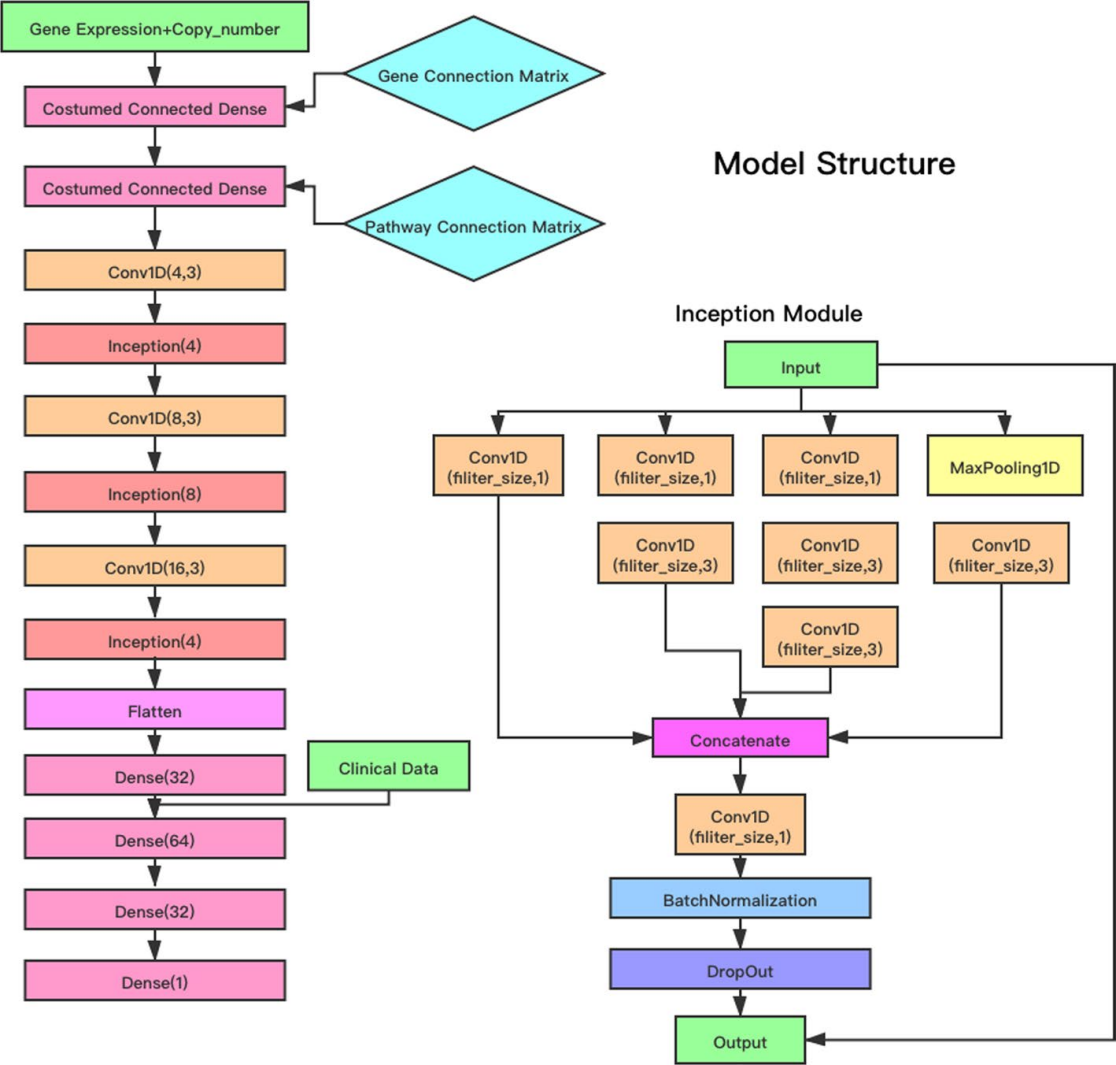
Schematic architecture of the DeepSigSurvNet model

Figure 1 shows the schematic architecture of the proposed *DeepSigSurvNet* model. In the ‘input layer’, there were two input features, i.e., normalized gene expression across TCGA samples and integer copy number variation, for each gene. Genes that have zero expression among training dataset will be excluded from input. In the model, gene expression and copy number variation information were first linked to individual genes to compute gene state respectively for each gene. Then, the genes’ state were connected to the 46 signaling pathways only if a gene was included in a signaling pathway (not a full connection layer). The gene connection matrix and pathway connection matrix were used to design the connections. The output of the 46 signaling pathways was used as the input for the convolution and inception [21] layers (see Fig. 1). The inception [21] module used multiple kernel filter sizes in each layer, instead of stacking more layers sequentially. It can capture informative features via the dimension reduction and reduce the vanishing gradient problem. The activation functions for the dense and convolution layers are the ReLU activation function. The last dense layer uses a linear activation function. To better model and predict the survival time of cancer patients, three clinical factors (age, gender and stage) and the vital status were concatenated with the genomics data. To reduce overfitting effects, the dropout layer and L2 weight decay were added in each inception module and the dense layer. For the training parameters, the batch size was 32 and the optimizer was “Adadelta”. The loss function is mean square error between the real survival time and predicted survival time. We divided the cancer samples in each type of cancer into training data (80%) and test data (20%). For the four cancer types, we used the same model architecture with a different dropout rate, regularization value, and epoch. After each epoch, we will evaluate the performance of model, the model parameter with the best test c-index will be recorded. To investigate the relevance of individual signaling pathways in survival time prediction, we employed the smoothgrad approach, which is available in the “iNNvestigate” package [22]. Specifically, noise signals or perturbations would be added to individual signaling pathways, and corresponding changes on the model prediction accuracy will be calculated. The gradient of the prediction accuracy changes for each individual signaling pathways (features) can be calculated and smoothed to indicate their influence to the survival time prediction. For the noise scale, we adjust it based on the input. To be more specific, $noise scale = (\max \left( {input} \right) - {\text{min}}\left( {input} \right))*0.1$.Then the distributions of the relevance scores of all 46 signaling pathways for each type of cancer were estimated using kernel density estimation based on the relevance scores of all samples and were obtained in order to investigate and understand the relevance of individual signaling pathways to the patients’ survival.

## Results

### Model performance evaluation

To evaluate the performance of the proposed model, the concordance index (c-index) metric was used. The c-index is defined as follows. Let $y_{i}$ and $\widehat{y}_{i} > \widehat{y}_{j} \left| {y_{i} > y_{j} } \right.$ be the true and predicted survival time. The concordance is defined as $P(\widehat{{y_{i} }} > \widehat{{y_{j} }}\left| {y_{i} } \right\rangle y_{j} )$, where *i* and *j* are two randomly selected samples. The c-index indicates the probability that the prediction and the real survival time are relatively consistent or concordant, i.e., $\widehat{{y_{i} }} > \widehat{{y_{j} }}, \,and\, y_{i} > y_{j}$, or $\widehat{{y_{i} }} < \widehat{{y_{j} }}, \,and\, y_{i} < y_{j}$. Let C, D, T represent the numbers for the concordant, discordant, and equal survival times, then the c-index is defined as:

$$c{ - }index = \frac{{C + \frac{1}{2}T}}{C + D + T}.$$

We compared the proposed model with the random forest model, which is available as RandomForestRegression in the scikit-learn package. We trained the random forest model using the same training and test dataset settings for the four types of cancer. The “n_estimator” and “max_depth” parameters were fine-tuned to find the best performance of the random forest models. For the DeepSigSurvNet model, we used the same architecture for all four types of cancer, but different dropout rates, regularization values and epoch numbers for each cancer type. Tables 3 and 4 show the comparison results. As shown, the random forest model has higher c-index values in the training datasets. However, it has much lower c-index values on the test datasets compared with the proposed DeepSignSurvNet model, which indicates that the proposed deep learning model is robust.

**Table 3:** C-index values of random forest model in four types of cancer

Data set	n_estimator	Max_depth	c-index
Training-GBM	30	5	0.6550
Test-GBM	30	5	0.5598
Training-BRCA	40	7	0.7849
Test-BRCA	40	7	0.5946
Training-LUAD	30	6	0.7433
Test-LUAD	30	6	0.5593
Training-SKCM	60	9	0.9419
Test-SKCM	60	9	0.5112

**Table 4:** C-index values of DeepSigSurvNet in four types of cancer

Data set	Epoch number	c-index
Training-GBM	35	0.6808
Test-GBM	35	0.6274
Training-BRCA	35	0.7930
Test-BRCA	35	0.6013
Training-LUAD	30	0.8263
Test-LUAD	30	0.7438
Training-SKCM	20	0.8103
Test-SKCM	20	0.7627

Considering the heterogeneity in TCGA dataset, multiple sampling at different ratios were also performed for evaluation of robustness. Specifically, we have tested different ratios of training data varying from 50, 60, 70, 80 and 90%, and repeated 50 times. The epoch time was set to 25, and the average c-index value of the 50 testing was used. The results are shown in Table 5. As seen, larger training dataset and small testing data have relatively better c-index values in the testing data. Overall, the proposed model outperformed the random forest on the testing data, though random forest model had better performance on the training data.

**Table 5:** Average c-index values of the proposed model and random forest model using different amount of training data. The mean c-index was obtained by randomly selecting the training and test dataset 50 times

Ratio of training data (%)	Proposed model		Random forest model	
	Mean c_index on training data	Mean c_index on test data	Mean c_index on training data	Mean c_index on test data
*GBM*				
50	0.6672	0.5869	0.7202	0.5383
60	0.6630	0.6033	0.7034	0.5404
70	0.6745	0.6029	0.6982	0.5450
80	0.6568	0.6085	0.6936	0.5493
90	0.6636	0.6392	0.6929	0.5612
*SKCM*				
50	0.7959	0.6961	0.7975	0.5178
60	0.7576	0.6680	0.7879	0.5237
70	0.7209	0.6950	0.7751	0.5277
80	0.7685	0.6717	0.7653	0.5255
90	0.7246	0.6643	0.7541	0.5208
*BRCA*				
50	0.6235	0.5435	0.8356	0.5217
60	0.6723	0.5768	0.8298	0.5262
70	0.6942	0.5627	0.8222	0.5154
80	0.7038	0.5835	0.8075	0.5026
90	0.7076	0.6157	0.8069	0.5036
*LUAD*				
50	0.7043	0.5857	0.8486	0.5641
60	0.7345	0.6708	0.8294	0.5702
70	0.6954	0.6363	0.8187	0.5704
80	0.7560	0.7249	0.8088	0.5809
90	0.7394	0.7419	0.8020	0.5822

To further test the influence of the number of pathways, the model was tested using the 10, 20, 30 and 40 signaling pathways. Specifically, a number of signaling pathways, e.g., 10 signaling pathways, were randomly selected for 50 times, using the 80% of the data as training and 20% of the data as the test data. The average c-index values were listed in Table 6. As can be seen, more signaling pathways achieved better c-index values in the training data and testing in general. The proposed model had better performance than the random forest model on the testing dataset. The results indicated that a small set of cancer signaling pathways are strongly informative for the cancer survival time prediction. It might be because that many of the cancer signaling pathways are overlapping and interact with each other. The random forest model had much better performance on the training data. However, it had poor performance on the testing data, which might be caused by the overfitting.

**Table 6:** Average c-index values of the proposed model and random forest model using different numbers of randomly selected signaling pathways. The mean c-index was obtained by randomly selecting the training (80% of the dataset) and test (20% of dataset) data for 50 times

# of pathway	Proposed model		Random forest model	
	Mean c_index on training data	Mean c_index on test data	Mean c_index on training data	Mean c_index on test data
*GBM*				
10	0.6428	0.6115	0.6832	0.5182
20	0.6442	0.6129	0.672	0.5159
30	0.6247	0.5975	0.6617	0.5314
40	0.6300	0.6196	0.6602	0.5418
*SKCM*				
10	0.6948	0.6626	0.7684	0.4943
20	0.7226	0.6702	0.7701	0.4434
30	0.7197	0.6614	0.772	0.4336
40	0.7629	0.6772	0.7739	0.4265
*BRCA*				
10	0.4397	0.3713	0.7859	0.5294
20	0.6051	0.4577	0.8029	0.5225
30	0.6661	0.5622	0.81	0.5282
40	0.6497	0.5506	0.8093	0.5139
*LUAD*				
10	0.7349	0.7050	0.8158	0.5195
20	0.7226	0.7131	0.8162	0.5349
30	0.7347	0.7171	0.8101	0.5551
40	0.7119	0.7226	0.8091	0.5669

### Relevance of individual signaling pathways in the four types of cancer

**Fig. 2:**
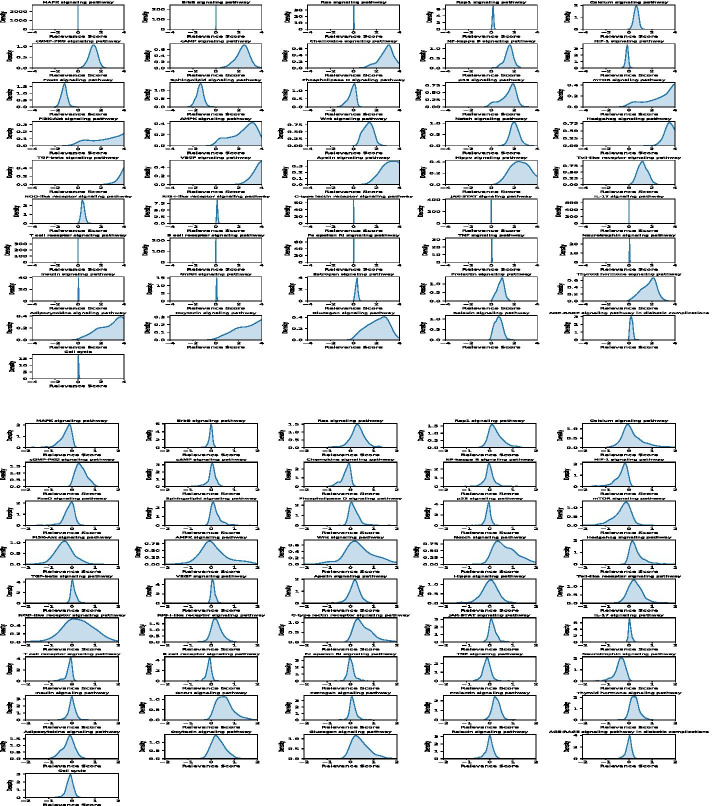
Density distribution of the relevance scores of the 46 signaling pathways on BRCA (top) and LUAD (bottom) cancers

**Fig. 3:**
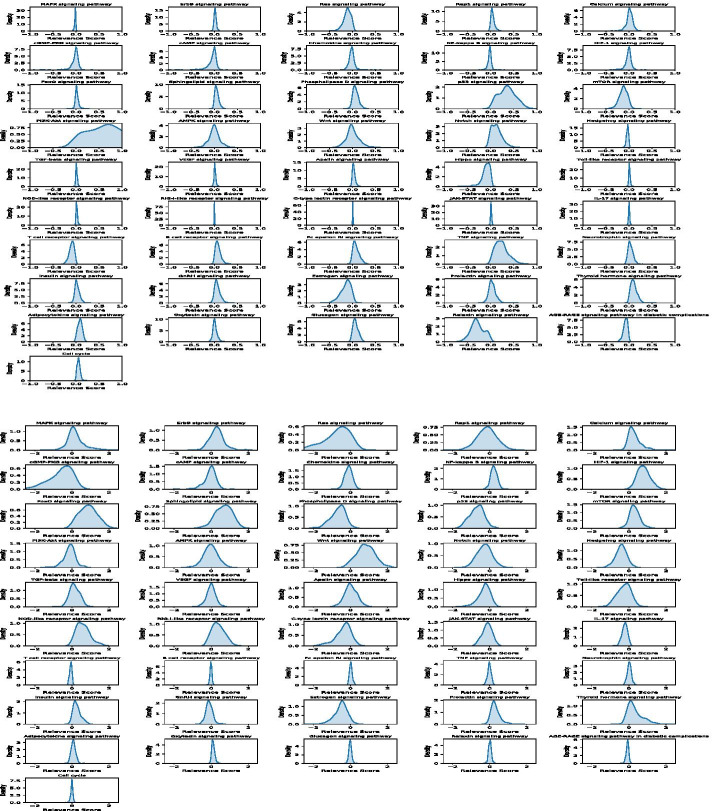
Density distribution of the relevance scores of the 46 signaling pathways on GBM (top) and SKCM (bottom) cancers

As discussed, it is interesting to investigate and understand how the individual signaling pathways contribute to the cancer patients’ survival prediction. After training the deep learning models, we employed the ‘iNNvestigate’ package to calculate the relevance scores of the individual signaling pathways on individual cancer patients in each of the four types of cancer. Figures 2 and 3 show the probability density distributions of 46 signaling pathways in the four types of cancer.

Specifically, the BRCA, mTOR, Hedgehog, PI3K-Akt, TGF-beta, AMPK, VEGF, Apelin, Adipocytokine and Oxytocin signaling pathways have the strongest relevance scores. P53, Wnt, Notch, NF-Kaapa B, FoxO, cGMP-PKG, cAMP, Chemokine, Sphingolipid, Relaxin, and Thyroid hormone signaling pathways have relatively high relevance scores. Surprisingly, the MAPK, ErbB, Ras, Rap1, and JAK-STAT signaling pathways as well as the cell cycle are not well associated with patients’ survival outcome, even though it is well known that these signaling pathways play important roles in cancer development. However, they can be separated in BRCA cancer samples and identified as the essential signaling pathways for patients’ survival outcome prediction. For LUDA, the patterns of density distribution are different from BRCA. More signaling pathways show high but not very strong relevance scores. For example, the MAPK, Ras, Rap1, cGMP-PKG, HIF-1, mTOR, PI3K-Akt, Wnt, Notch Hedgehog, C-type lectin receptor, GnRH, Neurotrophin, and Thyroid hormone signaling pathways have relatively high and consistent relevance scores. On the other hand, the AMPK, Hippo and NOD-like signaling pathways have zero-mean values but with great variance. Thus, it is hard to evaluate their relative importance in cancer patients’ survival prediction analysis. For GBM, the Ras, p53, mTOR, PI3K-Akt, Notch, Hippo, TNF, Estrogen, Thyroid hormone and Relaxin signaling pathways have relatively high relevance scores; the other signaling pathways are not correlated with patients’ survival. For SKCM, the patterns are similar to the LUAD cancer samples. The Ras, Calcium, cGMP-PKG, NF-Kappa B, HIF-1, FoxO, Sphingolipid, Phospholipase D, p53, mTOR, Wnt, Hedgehog, NOD-like receptor, Estrogen, Prolactin, and Thyroid hormone signaling pathways have relatively high and consistent relevance scores. Whereas, the MAPK, Rap1, PI3K-Akt, AMPK, and VEGF signaling pathways have zero-mean values but with great variance.

In summary, the probability density distribution patterns of all 46 signaling pathways vary significantly among the four types of cancer. For example, the p53 and mTOR signaling pathways are strongly relevant to patients’ survival outcomes in BRCA, GBM, and SKCM cancer patients, but not in the LUDA cancer patients. The MAPK, RAS, Rap1, and ErBB signaling pathways are known as the important signaling pathways in cancer, but they are not strongly correlated with cancer patients’ survival outcome in the prediction models. This might be because all of these important signaling pathways are always activated in cancer patients. Thus, they are important targets for cancer therapy, but not informative in terms of the survival time prediction. Also, the cell cycle signaling does not play an important role in the survival time prediction. Moreover, a small set of signaling pathways (e.g., T cell receptor, B cell receptor, Fc epsilon RI, TNF) do not show important contributions to the survival of cancer patients across all four types of cancer. Also, for each type of cancer, less than half of the signaling pathways have strong effects on the survival prediction. Thus, drugs and drug combinations that can inhibit these essential signaling pathways as well as the signaling pathways with strong relevance scores for each type of cancer might be effective in improving cancer patients’ survival time and outcome.

## Discussion

Survival prediction is important in cancer studies. Deep learning models that integrate multi-omics data have been proposed for survival prediction and have outperformed the classic Cox PH model. Signaling pathways are important in cancer research to understand the signaling cascades regulating cancer development and drug response. However, it is challenging to understand the contributions of individual genes considering the non-linear combinations of a large number of genomic features, e.g., gene expression, copy number variation. Instead of using a large number of genomics features, in this study, we proposed a relatively biologically meaningful and simplified deep learning model, DeepSigSurvNet, for survival prediction. In the model, the gene expression and copy number data of 1967 genes from 46 major signaling pathways were used. The deep learning model analysis on four types of cancer can identify the distinct patterns of these signaling pathways, which are helpful in understanding the relevance of the signaling pathways in the context of survival analysis. These pathways can also be novel targets for drug and drug combination prediction to improve cancer patients’ survival outcome.

There are some improvements to the proposed model that need to be further investigated. In addition to the 46 signaling pathways, other KEGG pathways, like metabolism pathways, will be further evaluated. Moreover, Gene oncology [23] (GO) terms provide alternative meaningful biological processes (BP) (gene sets). Moreover, cancer subtype information is often related to different survival patterns. Identification and incorporation of the subtype information can be useful to improve the model. In addition, validation using independent datasets is necessary in order to evaluate the generalizability of the model. Other omics data such as protein, methylation, and genetic mutation can be conveniently integrated into the model in addition to the copy number, gene expression data. As aforementioned, the important genes within the important signaling pathways can be used as potential gene signatures to discover drugs using the connectivity map (CMAP) [24, 25]. In this study, the proposed model is partially biological meaningful due to the use of signaling pathways. However, the detailed signaling structure information has not been modeled. The deep graph neural network (GNN) could be used to better model the signaling structure, i.e., cascade connections. We will investigate these possible directions in future work.

## Conclusion

In this study, we proposed a biologically meaningful and simplified deep learning model, DeepSigSurvNet, based on a set of signaling pathways to model cancer patients’ survival. Multi-omics data and clinical factors can be integrated into the model in a relatively meaningful manner compared with existing deep learning models, and the model is robust for testing data. The interpretable analysis can help researchers understand the effects of individual signaling pathways and identify new therapeutic drugs that target the top correlated signaling pathways relevant to patient survival time and outcome.

## Data Availability

Data availability is provided in Table I.

## Abbreviations

NLP: Nature language process
ANOVA: Analysis of variance
TCGA: The Cancer Genome Atlas
GSEA: Gene set enrichment analysis
SCNN: Survival Convolutional Neural Networks
BRCA: Breast Invasive Carcinoma
LUAD: Lung adenocarcinoma
GBM: Glioblastoma multiforme
SKCM: Skin cutaneous melanoma
LRP: Layer-wise relevance propagation
KEGG: Kyoto Encyclopedia of Genes and Genomes
CMAP: Connectivity map
